# The Effects of Turnip (*Brassica rapa*) Extract on the Growth Performance and Health of Broilers

**DOI:** 10.3390/ani11030867

**Published:** 2021-03-18

**Authors:** Reza Eghbaldost-Jadid, Mehran Nosrati, Behrouz Rasouli, Alireza Seidavi, Clive J. C. Phillips

**Affiliations:** 1 Department of Animal Science, Rasht Branch, Islamic Azad University, Rasht 4135-3516, Iran; arash.eghbaldoust@gmail.com (R.E.-J.); nosrati@iaurasht.ac.ir (M.N.); brasooli@gmail.com (B.R.); 2 Curtin University Sustainable Policy (CUSP) Institute, Curtin University, Kent St., Bentley, Western Australia 6102, Australia

**Keywords:** growth promoters, meat chickens, antibiotics, antibodies

## Abstract

**Simple Summary:**

Antibiotics are commonly added to the diet of chickens grown for meat to reduce bacterial contamination of their gastrointestinal tract. The bacteria reduce the efficiency of feed utilization and, hence, growth. However, there are concerns about the inclusion of antibiotics in the feed of chickens grown for meat, because of the development of resistance in the bacteria. As a result, scientists are searching for alternative feed additives. Turnip extract is known to have antibacterial properties but has not been tested in the diet of broiler chickens. We tested several levels of turnip extract in the water for chickens and compared their growth and the level of bacterial contamination of their gut with that of chickens given a standard antibiotic. Although chickens with the highest level of turnip extract initially had slow growth, those given a medium level of turnip extract had faster growth overall, better feed conversion, fewer Gram-negative lactose bacteria in their cecum and fewer antibodies in their blood, compared with those fed the antibiotic. This suggests that inclusion of turnip extract in the diet of chickens could provide an alternative to conventional antibiotics.

**Abstract:**

There are concerns about inclusion of antibiotics in the feed of broiler chickens, because of the development of antibiotic resistance, leading to a search for alternative feed additives. Turnip extract is known to have antibacterial properties but has not been tested in the diet of broiler chickens. We allocated 200 broiler chicks to receive one of four levels of turnip extract in their water, 0, 150, 300 or 450 ppm, or a standard antibiotic, Virginiamycin, over a 42-day growing period. Although initially there were detrimental effects of providing 450 ppm, overall the 150 ppm level of supplementation increased weight gain, compared with birds given Virginiamycin, and decreased gizzard weight. Birds given 150 ppm or Virginiamycin had increased low-density lipoproteins (LDLs) and reduced very low-density lipoproteins (VLDLs) in their blood serum and reduced antibody responses to sheep red blood cells, compared to birds in the 450 ppm treatment. Birds given turnip extract at 450 ppm had fewer Gram-negative lactose and coliform bacteria than those provided with no turnip extract, and those provided with 150–300 ppm had the same as those provided with Virginiamycin. Turnip extract could potentially replace antibiotics included in the feed of broiler chickens for growth promotion and the control of bacterial infection of the gastrointestinal tract.

## 1. Introduction

Scientists are searching worldwide for feed and water additives that improve chicken’s health and performance, following evidence of resistance and cross resistance to antibiotics in pathogenic bacteria [1]. Of greatest concern is transmission of resistance to *Escherichia coli*, which has been observed from poultry to humans [2]. Although it has been argued that emerging antibiotic resistance is more a result of overuse of antibiotics to control human diseases [3], consumer demand for alternatives is strong, and hence it is important to find novel feeds that will improve chicken health and performance. Virginiamycin is commonly fed to broiler chickens at either growth promoting concentrations (c. 11 ppm) or prophylactically at higher doses to control diseases, such as necrotic enteritis arising from *Clostridium perfingens* colonization of the gastrointestinal tract, particularly in the ileal region [4].

One possible phytogenic additive is extract of turnip (*Brassica rapa*). The turnip, or white turnip (*Brassica rapa* subsp. rapa), is a root vegetable commonly grown in temperate climates for its white, fleshy taproot, and one of the world’s oldest cultivated vegetables [5]. The word turnip is a compound of “tur”, as in turned/rounded on a lathe, and “neep”, derived from Latin napus, the word for the turnip plant. Small, tender varieties are grown for human consumption, while larger varieties are grown as feed for livestock. Turnips are an excellent source of bioactive compounds, which potentially deliver health benefits to animals when consumed. These include peroxidases, kaempferol, phenolic compounds, sulforaphane, organic acids, vitamin K and glucosinolates [6]. The claimed biological activities are many, including anti-oxidant, anti-tumor, anti-diabetic, anti-inflammatory, anti-microbial, hypolipidemic, cardioprotective, hepatoprotective, nephroprotective and analgesic capabilities. The antimicrobial activity arises from its content of phenols and flavonoids, particularly when the latter are concentrated by ethyl acetate extraction [7,8]. Extraction of the active ingredients potentially offers antimicrobial activity of benefit to the food industry [9]. The potency of the various extraction methods for different plant components has been quantified as follows:

aqueous extract of roots *>* aqueous extract of turnip greens *>* ethanol extract of roots *>* light petroleum fraction of roots *>* ethanol and ethyl acetate extract of turnip greens *>* chloroform extract of roots [10].

There are no reports of the effects of offering turnips or their extracts to chickens. Existing antibacterial treatment of broiler chickens with antibiotics is creating a risk of resistance developing, hence there is a search underway for phytochemicals that will have similar beneficial effects on growth and health of the birds. Turnip extract has been accredited with antioxidant and antibacterial properties in other species, but these are unexplored in chickens. Therefore, this study tested the hypothesis that dietary turnip extract will improve the performance of broiler chickens during a 42-d production cycle. The performance was evaluated based on growth, carcass characteristics, blood biochemistry, immunity and cecal microbiota under varying levels of supplementation with turnip extract.

## 2. Materials and Methods

This study was carried out on a commercial poultry farm at Guilan, Iran. The experimental protocol was ratified by the Animal Ethic Committee of the Islamic Azad University (Approval number 117503972003), and the experiment performed in accordance with the International Guidelines for research involving animals (Directive 2010/63/EU).

A total of 200 Ross 308 broiler chicks were purchased and raised under thermo-neutral ambient temperatures, according to the standard brooding practices for the rearing stages of the birds [11]. The birds received 24 h lighting on day (d) 1, and 23 h subsequently. Broiler diets were formulated for 3 growth phases (starter: day 1 to 14; grower: day 15 to 28 and finisher: day 29 to 42; Table 1). The birds were vaccinated against influenza on day 1, infectious bronchitis on days 1 and 8, Gumboro Disease on days 16 and 32, and Newcastle Disease on days 1 and 8.

**Table 1:** Ingredients, chemical composition and metabolizable energy of the starter, grower and finisher diets ^1^.

	Starter (Day 1 to 14)	Grower (Day 15 to 28)	Finisher (Day 29 to 42)
**Ingredients (%)**			
Corn	54.5	58.5	62.7
Soybean meal	37.5	33.2	29.5
Soybean oil	4.0	4.0	4.0
Calcium carbonate	1.2	1.2	1.1
Dicalcium phosphate	1.6	1.5	1.5
Salt (NaCl)	0.23	0.26	0.25
Vitamin and mineral premix ^1^	0.6	0.6	0.6
Sodium bicarbonate	0.12	0.14	0.1
DL-Methionine	0.18	0.21	0.15
L-Lysine	0.07	0.09	0.1
**Chemical Composition**			
Metabolisable energy ^2^ (kcal/kg)	3010	3050	3100
Crude protein (%)	21.04	19.60	18.18
Lysine (%)	1.27	1.10	0.97
Methionine + Cystine (%)	0.94	0.84	0.76
Methionine (%)	0.47	0.42	0.36
Arginine (%)	1.31	1.14	1.02
Tryptofan (%)	0.20	0.18	0.16
Calcium (%)	1.05	0.90	0.85
Available phosphorus (%)	0.50	0.45	0.42
Magnesium (%)	0.05	0.06	0.05
Sodium (%)	0.21	0.19	18.18
Chloride (%)	0.17	0.17	0.16
Potassium (%)	0.50	0.40	0.40
Copper (mg/kg)	16.0	16.0	18.0
Iodine (mg/kg)	1.25	1.25	1.25
Iron (mg/kg)	40.0	40.0	40.0
Manganese (mg/kg)	120.0	120.0	120.0
Selenium (mg/kg)	0.30	0.30	0.30
Zinc (mg/kg)	100.0	100.0	100.0
Vitamin A (IU/kg)	11000	9000	9000
Vitamin E (IU/kg)	75	50	50
Vitamin K (mg/kg)	3	3	2
Vitamin B_12_ (mg/kg)	0.016	0.016	0.010
Riboflavin (mg)	8	6	5

^1^ Amount per kg: vitamin A, 5000 IU; vitamin D_3_, 500 IU; vitamin E, 3 mg; vitamin K_3_, 1.5 mg; vitamin B_2_, 1 mg; calcium pantothenate, 4 mg; niacin, 15 mg; vitamin B_6_, 13 mg; Cu, 3 mg; Zn, 15 mg; Mn, 20 mg; Fe, 10 mg; K, 0.3 mg. ^2^ Metabolic availability (from UFFDA output [12]). ^2^ Metabolizable energy was estimated using the Carpenter and Clegg equation [13].

The birds were allocated on d1 of age to 20-floor pens (each 1 × 1 m) containing 10 birds each, five females and five males. Wood shavings were provided as bedding. Birds in 4 replicate pens received 0, 150, 300 and 450 ppm turnip extract in drinking water from days 1–42 based on a completely randomized design. Inclusion rates were based on a previous study of antimicrobial activity [14], in which the strongest inhibition of fungi was by turnip root extract included at 100 ppm, but it was noted that protective effects were dose dependent. Four replicate pens of a control treatment with no turnip extract but incorporating a Virginiamycin antibiotic at 200 g/tonne were also included, a high therapeutic dose rate to achieve maximum effect. Alcohol-extracted turnip was purchased from the Institute for Medicinal Plants Research of Academic Jihad (Karaj, Iran) (http://imp.acecr.ac.ir, accessed on 10 March 2021). Production of turnip extract followed the method of Kim et al. [15] and involved cutting 1 kg fresh roots, macerating them, boiling in 1.2 L ultrapure water 3 times for one hour each, extracting 3 times with 95% ethanol (1 L each time) and centrifuging at 3000 rpm to obtain the crude polysaccharide extract. This was then washed in ethanol until the filtrate ran pure, after which the extract was spun-dried at 40 °C using a rotatory evaporator at low pressure. The yield after vacuum evaporation was 10.6 g per 100 g of fresh root material.

Provision of feed and water were ad libitum during the 42 d growing period. Body weight and feed intake (the difference between offered and refused feed) were measured weekly. Average daily feed intake, average daily weight gain and feed conversion ratio were calculated for each replicate within each treatment for the starter period, grower period, finisher period, and whole study period (day 1 to 42). An economic index was calculated as follows: Economic index = (Weight at d 42 (g) × 10)/(Feed Conversion Ratio from d 1–42 × 42).

On the last day of the experiment (day 42), one bird per pen was chosen randomly to evaluate the characteristics of the carcass. It was removed from each pen and weighed, then, after 4 h without food or water, it was killed by cervical dislocation. The weights of the entire defeathered carcass and of the carcass without head and drumsticks were recorded. Viscera and abdominal fat were then removed, and the carcass yield and weights of abdominal fat, anatomical parts (breast, drumsticks, wings and neck), and empty gut tract components (duodenum, jejunum, ileum, colon, right and left cecum and rectum) were determined. Additionally, on day 42, 1 broiler from the remaining birds in each pen was chosen at random to evaluate immune responses. They were killed by cervical dislocation, and the relative weights of organs relevant to the immune system function (spleen and cloacal bursa) were calculated according to Shabani et al. [16].

At day 42, blood samples (1.5 mL) were collected from two of the remaining birds in each pen (n = 40) into EDTA tubes (Merck, New York, NY, USA) from a wing vein. Blood serum was obtained by centrifugation (Rotofix 32A centrifuge, Hettich, Germany; 1500× *g* for 10 min) and chilled at –18 °C until analysis. The levels of uric acid, total cholesterol, triglycerides, high, low and very low density lipoproteins (HDLs, LDLs and VLDLs, respectively), glucose, total protein, and albumin, were measured using commercial laboratory kits (Pars Azmoon Co., Tehran, Iran [17]).

Two additional birds per replicate (n = 40, chosen to have a weight most similar to the replicate average) on days 14 and 35 were selected to evaluate the systemic antibody response [5]. Birds were vaccinated against sheep red blood cells (SRBCs) by subcutaneous administration of SRBC suspension in 5% phosphate-buffered saline (PBS). At 28 and 42 days of age, blood samples were collected after 4 h without food and water and pooled within replicates, and total Ig against SRBCs was determined using a hemagglutination assay in serum 7. In U-bottom microtiter plates, two-fold serial dilutions of heat-inactivated serum (at 56 °C) were added to PBS (0.01 mol/L; pH 7.4) for assessment of total antibodies, and to PBS with 1.4% 2-mercaptoethanol for assessment of IgG antibodies. All antibody titers were recorded as log_10_ of the highest dilution of serum that agglutinated an equal volume of a 0.5% SRBC suspension in PBS [18]. The IgM titer was determined as the difference between the total titer and the IgG titer.

Cecum microbiota counts were based on the technique described by Dibaji et al. [18]. Briefly, agar plates were streaked with randomly sampled cecal contents, with the aim of determining the bacterial growth and colony counts. The sample collection tubes were weighed and wrapped in an aluminum sheet before processing in an autoclave for 10 min. The culture media were prepared and poured into the Petri dish 24 h before sample collection. The collection tubes were weighed empty and full and the weight of the sample in each tube obtained as the difference between the two values. The tubes were shaken for about 30 min. This was done separately for bacteria isolated from gastrointestinal contents and suspension preparation. About 1 ml of the resulting suspension was collected and mixed with 9 mL PBS in the other tube. Lactobacilli bacteria were incubated at 37 °C under anaerobic conditions for 72 h. The bacteria count was taken using a colony counter. The bacterial counts were obtained as the logarithm of bacteria number per gram of the sample.

### Statistical Analysis

Data was analyzed for one-way ANOVA using the GLM procedure of IBM SPSS Statistics 21 software for Windows^®^ [19]. Pen means were the experimental units for growth parameters, whereas carcass characteristics, blood constituents, immune response and gut microbiota were analyzed using the results of the individual bird in each replicate used for each measurement. Post hoc pairwise comparisons were by Duncan’s test. Significant differences between group means were reported at *p* ≤ 0.05.

## 3. Results

There was no effect of treatment on weight gain in the Starter period (Table 2), even though there was a reduction in the 450 ppm treatment in week one compared with all of the other treatments (Appendix A Table A1). Birds in the 450 ppm treatment increased their feed conversion ratio in the first week of the starter period but reduced it in the second week (Appendix A Table A1), when compared with the Virginiamycin treatment. In the grower period, weight gain was highest in the 0 ppm treatment, but there were no differences between treatments in feed intake or feed conversion ratio. In the finisher period, weight gain was higher in the 150 ppm than the 0 and 450 ppm treatments and least in the Virginiamycin treatment. Feed intake was higher in the 0 and 150 ppm treatments than the 450 ppm treatment, and feed conversion ratio greater in the 150 and 300 ppm treatments than the 0 and Virginiamycin treatments. Over the entire experiment, weight gain was higher in the 150 ppm treatment than the Virginiamycin treatment and feed intake tended to be higher in the 0 and 150 ppm treatments than the 450 ppm treatment. Feed conversion ratio was increased for birds in the 0 ppm and Virginiamycin treatments, compared with the other three treatments.

In the starter period, the economic index was increased for birds offered the 150 ppm treatment, compared with those offered the Virginiamycin treatment, but in the grower period it was higher for the 0 and 450 ppm treatments. In the finisher period, the economic index was higher in the 150 and 300 ppm treatments than the 0 or Virginiamycin treatments. Overall, the economic index was higher for the 150 and 300 ppm treatments compared with the Virginiamycin treatment.

There were no significant effects of treatment on empty abdominal carcass, breast, drumstick, wings, heart or liver weights at 42 d of age (Table 3). However, abdominal fat was higher in the 300 ppm treatment than the 450 and 0 ppm treatments. Gizzard weight was less in the 150 ppm treatment than 300, 450 and Virginiamycin treatments.

There were no significant treatment effects on blood serum glucose, HDLs or total cholesterol, although the cholesterol content tended to be increased in the Virginiamycin treatment (*p* = 0.09, Table 4). Triglycerides were higher in the 450 ppm treatment than 150 or Virginiamycin. LDLs were lower at 300 and 450 ppm, compared with 150 and Virginiamycin, and VLDLs higher at 450 ppm, compared with all other treatments.

At 28 d of age, in response to sheep red blood cells, IgG was reduced in the 150 ppm treatment, compared with the other turnip extract treatments, IgM was increased in the 450 ppm treatment, and least in the Virginiamycin treatment, and total Ig was increased in the 450 ppm treatment and least in the 150 ppm treatment (Table 5). At 42 d of age, there were no effects of treatment on IgG or IgM responses, but at 150 ppm of turnip extract total Ig was reduced compared with the other treatments. The weight of the spleen was higher in the Virginiamycin treatment compared with the 0 ppm treatment, but there were no treatment effects on the weight of the cloacal bursa (Table 6). Coliform bacteria were more numerous in the Virginiamycin treatment and least in the 450 pm treatment. The cecal Gram-negative lactose bacteria were greatest for the 0 ppm treatment, tended to decline with increased turnip extract and were least in the Virginiamycin treatment (Table 7).

## 4. Discussion

The study addressed the effects of alcohol-extracted turnip on the health and performance of broiler chickens. In the 450 ppm treatment, the negative effect on weight gain in the first week, followed by reduced intake, suggests an antinutritional impact in the early stages of feeding the turnip extract that was unrelated to palatability. No adverse effects on intake of feeding turnips to pigs been recorded [20]; however, reduced intakes have been noted in sheep [21]. There are potentially negative effects of glucosinolates from turnips on performance; in particular their post-ingestional derivatives may interfere with iodine uptake and the production of triiodothyronine (T3) and thyroxine (T4) [22]. This may lead to hypothyroidism and goiter. In cattle, glucosinolates from turnips are reported to cause photosensitization following liver damage [23]. In poultry glucosinolates reduce intake and performance, but effects are more severe in laying hens and turkeys than broilers [24]. The glucosinolates that interfere with thyroid activity may have been responsible for this, but it is unclear if this could be manifested within a week. This antinutritional effect appears to have continued into the Grower period, since weight gain was highest in the 0 ppm treatment for this period. In the Finisher period some benefit of the 150 ppm level of inclusion was apparent, with higher growth rates in this period for this treatment, and overall.

Feed conversion was least efficient for birds in the 0 ppm and Virginiamycin treatments, again suggesting some benefit of inclusion of turnip extract. Virginiamycin has been found in a meta-analysis of poultry studies to improve feed conversion on average by about 4% when included in their diet [25]. It is likely that our study did not have enough replication to detect the small improvement detected in the meta-analysis, which included a total of 121,643 broilers. The benefits of inclusion of turnip extract on feed conversion were not dependent on dosage and may derive from the high vitamin K content.

The reduction in LDLs in the 450 ppm treatment, in comparison to the Virginiamycin treatment, may be attributed to an improved antioxidant status, which has been reported in rats supplemented with turnip extract [15]. LDLs and VLDLs are two of the five major groups of lipoproteins that enable lipids, including cholesterol and triglycerides, to be transported in the blood. The increase in abdominal fat and reduction in LDLs in the birds receiving 300 ppm turnip extract requires further research. It may derive from improved antioxidant status, preventing the LDLs from oxidizing to lipid hydroperoxides. These are unstable and could lead to abdominal fat deposition. This is likely to be a dynamic process and following fat deposition the LDLs may be depleted. Research in humans has suggested that turnip oil will reduce LDLs cholesterol by interfering with cholesterol metabolism [26]. The tendency for a reduction in serum cholesterol with turnip extract inclusion supports this. However, the simultaneous increase in triglycerides and VLDLs in the 450 pm treatment birds may cause increased risk of cholesterol deposits, which, if proven, suggests that there are dose-dependent responses. This might relate to the tendency for a reduction in feed intake over the whole study in the 450 ppm treatment.

There was some evidence of increasing antibacterial activity of the turnip extract with increasing concentration. Coliform bacteria in the cecum were reduced at the highest concentration and there was a gradual reduction in Gram-negative lactose bacteria with inclusion of turnip extract. There has been similar evidence of antibacterial activity of turnip in an infected mouse model, in which a high dose had anti-*Helicobacter pylori* activity [27]. In the latter study, there was also an increased anti-H. pylori IgG titer, and we observed increased IgG titers in the 450 ppm treatment, compared with the 150 ppm treatment.

Antibiotics are the conventional method of manipulating the bacterial composition of the gut microbiota, which varies with the stage of development of the chicken and the different sections of the gut. Manipulation of gut microbiota can be beneficial in terms of improved nutrient digestion and absorption, in particular of lipids and amino acids, and better immunity and health in the host. There are varied results obtained from investigations of the effects of antibiotics on gut microbiota, depending on the antibiotics used. In one study, neomycin and ampercillin increased bacteria of the phylum Firmicutes, but also some pathogenic bacteria (e.g., Rikenellaceae and Enterobacteriaceae) [28]. However, in another study [29], Bacteroidetes increased and Firmicutes decreased in response to Virginiamycin. Bacteroidetes ferment less digestible polysaccharides and increase fat accumulation in chickens and are generally considered beneficial to gut health in the chicken.

Coliforms were probably high in the Virginiamycin group of our study because the Gram-negative lactose bacteria were low. This may have led to spleen enlargement. Gram-negative lactose-digesting bacteria include *Escherichia coli*, fermenting lactose to produce hydrogen sulfide, whereas the coliform bacteria are generally not harmful. Virginiamycin is known to reduce Gram-negative-susceptible bacteria, including most *Haemophilus* species, *Neisseria gonorrhoeae* and *N. meningitidis* [30], but these are not lactose fermenting [31]. It also reduces lactobacilli, which have probiotic functions [4].

The low humoral Ig concentrations in chickens given the turnip extract may reflect low levels of antigen challenge. There is evidence that turnip extract increases, in a dose-dependent manner, both innate and acquired immunity in mice injected with sheep red blood cells [32]. There were probably no effects on the cloacal bursa because it withers as the bird grows, with older birds relying on the spleen and peripheral lymph nodes to sustain their immune response. Of all the turnip inclusion rates, from 0 to 450 ppm, the coliforms and Gram-negative lactose bacteria were least in the 450 ppm group. This suggests that the turnip extract may have had direct bactericidal effects, because the immunomodulation of antibody concentrations was least at 150 ppm, at which concentration there were moderate bacteria concentrations in the cecum.

## 5. Limitations

We acknowledge that the turnips selected for the preparation of extract may not have been representative of the species. Further studies are needed to evaluate the differences between different sizes of turnip, different cultivars, different fertilizer regimes and soil types, particularly recognizing the dose-dependent benefits observed in this study. It is hoped that these will be stimulated by the positive results demonstrated by the medium level of turnip extract inclusion in our study. It is also possible that the doses selected were not optimal for antimicrobial effects in the gastrointestinal tract of chickens, but we based the doses on the only relevant study [14], in which different doses of turnip root extract were tested on fungal plates. We tested both higher and lower doses than the optimal dose derived from this experiment. It is also possible that the replication of birds for microbiota and antibodies was not adequate, but we had an ethical obligation to minimize animal numbers, and since we obtained significant effects with the replication levels that we had, we conclude that the power of the experiment to detect significant differences was adequate.

## Figures and Tables

**Table 2:** Performance means ±SEM of Ross 308 broilers during the starter, grower, finisher and total periods when supplemented with different levels of *Brassica rapa* extract at 0–450 ppm or Virginiamycin from weeks 1–6 *.

	Weight Gain g/Period	Feed Intake g/Period	Feed Conversion Ratio **	Economic Index ***
Starter period at d 1–14				
Virginiamycin at 0.2 g/kg	429.4	519.5	1.21	254.2
Turnip extract at 0 ppm	431.0	492.1	1.15	270.8
Turnip extract at 150 ppm	451.8	490.1	1.08	298.1
Turnip extract at 300 ppm	442.5	489.7	1.11	286.3
Turnip extract at 450 ppm	439.2	481.3	1.09	286.5
*p*-value	0.54	0.21	0.1	0.21
Standard Error of Mean	4.46	5.401	0.0163	6.258
Grower period at d 15–28				
Virginiamycin at 0.2 g/kg	951.2 ^bc^	1358	1.42	476.3 ^b^
Turnip extract at 0 ppm	1007.8 ^a^	1355	1.34	537.2 ^a^
Turnip extract at 150 ppm	938.8 ^bc^	1327	1.41	474.5 ^b^
Turnip extract at 300 ppm	917.2 ^c^	1263	1.37	476.8 ^b^
Turnip extract at 450 ppm	976.8 ^ab^	1286	1.31	531.0 ^a^
*p*-value	0.003	0.22	0.16	0.02
Standard Error of Mean	8.99	15.66	0.0163	9.037
Finisher period at d 29–42				
Virginiamycin at 0.2 g/kg	1002 ^c^	1781 ^ab^	1.77 ^a^	403.3 ^b^
Turnip extract at 0 ppm	1036 ^bc^	1909 ^a^	1.85 ^a^	404.1 ^b^
Turnip extract at 150 ppm	1288 ^a^	1910 ^a^	1.49 ^b^	625.7 ^a^
Turnip extract at 300 ppm	1264 ^ab^	1820 ^ab^	1.46 ^b^	636.9 ^a^
Turnip extract at 450 ppm	1038 ^bc^	1650 ^b^	1.61 ^ab^	470.9 ^ab^
*p*-value	0.04	0.04	0.01	0.04
Standard Error of Mean	41.35	32.36	0.0471	34.85
Total period at d 1–42				
Virginiamycin at 0.2 g/kg	2383 ^b^	3658	1.53 ^a^	369.7 ^b^
Turnip extract at 0 ppm	2475 ^ab^	3756	1.51 ^a^	389.1 ^ab^
Turnip extract at 150 ppm	2678 ^a^	3728	1.39 ^b^	459.3 ^a^
Turnip extract at 300 ppm	2624 ^ab^	3573	1.36 ^b^	460.9 ^a^
Turnip extract at 450 ppm	2454 ^ab^	3417	1.39 ^b^	419.7 ^ab^
*p*-value	0.08	0.09	0.01	0.04
Standard Error of Mean	39.91	44.58	0.0219	12.29

* Means within each column without common superscripts are significantly different at *p* < 0.05. ** Feed intake/weight gain. *** Economic index = (d 42 Weight (g) × 10)/(Feed Conversion Ratio (d 1–42) × 42).

**Table 3:** Mean ±SEM of carcass and organs weights of Ross broilers at d 42 when supplemented with different levels of *Brassica rapa* extract at 0–450 ppm or Virginiamycin from weeks 1–6 *.

	Empty Abdominal Carcass	Breast	Drumsticks	Wings	Abdominal Fat	Heart	Liver	Gizzard
Virginiamycin at 0.2 g/kg	1940	710	548	56.5	40.2 ^ab^	12.2	63.7	44.0 ^a^
Turnip extract at 0 ppm	1747	655	508	53.2	34.5 ^b^	10.2	56.2	41.2 ^ab^
Turnip extract at 150 ppm	1765	694	538	55.7	41.5 ^ab^	13.7	53.7	34.5 ^b^
Turnip extract at 300 ppm	1722	653	474	57.0	52.2 ^a^	11.5	54.7	48.2 ^a^
Turnip extract at 450 ppm	1897	669	539	52.2	33.2 ^b^	13.7	50.0	45.7 ^a^
*p*-value	0.17	0.54	0. 06	0.84	0.02	0.06	0.15	0.02
SEM	34.83	12.14	9.63	1.47	2.17	0.47	1.77	1.52

* Means within each column without common superscripts are significantly different at *p* < 0.05.

**Table 4:** Mean ±SEM of blood serum parameters at 42 d of age in Ross 308 broilers supplemented with different levels of *Brassica rapa* extract at 0–450 ppm or Virginiamycin from weeks 1–6 *.

	Glucose mg/dL	Total Cholesterol mg/dL	Triglycerides mg/dL	HDLs mg/dL	LDLs mg/dL	VLDLs mg/dL	Albumin mg/dL	Total Protein mg/dL	Uric Acid mg/dL	LDL/HDL Ratio
Virginiamycin at 0.2 g/kg	211.5	150	81.0 ^b^	61.2	75.0 ^a^	16.2 ^b^	1.55 ^c^	4.86 ^bc^	5.19 ^b^	0.82 ^b^
Turnip extract at 0 ppm	223	143	92.0 ^ab^	61	62.1 ^ab^	18.0 ^b^	1.69 ^b^	3.47 ^d^	5.69 ^ab^	0.98 ^ab^
Turnip extract at 150 ppm	206.2	144.7	73.5 ^b^	61.5	68.6 ^a^	14.4 ^b^	1.75 ^b^	4.35 ^c^	5.88 ^a^	0.93 ^b^
Turnip extract at 300 ppm	206.2	131.7	94.7 ^ab^	62.2	50.6 ^b^	18.8 ^b^	1.84 ^a^	5.40 ^ab^	4.61 ^c^	1.25 ^a^
Turnip extract at 450 ppm	215	137.2	117.7 ^a^	61.7	51.1 ^b^	24.1 ^a^	1.70 ^b^	5.57 ^a^	5.41 ^ab^	1.23 ^a^
*p*-value	0.11	0.09	0.03	0.88	0.01	0.01	0.001	0.001	0.002	0.02
SEM	2.33	2.31	4.97	0.373	2.97	1	0.0249	0.193	0.123	0.053

* Means within each column without common superscripts are significantly different at *p* < 0.05. HDLs = high-density lipoproteins; LDLs = low-density lipoproteins; VLDLs = very low-density lipoproteins.

**Table 5:** Mean (log_10_) antibody response titers to sheep red blood cells at 28 and 42 d of age in Ross 308 broilers supplemented with different levels of *Brassica rapa* extract at 0–450 ppm or Virginiamycin from weeks 1–6 *.

	28 d of Age			42 d of Age		
	IgG	IgM	Total Ig	IgG	IgM	Total Ig
Virginiamycin at 0.2 g/kg	4.50 ^ab^	2.50 ^b^	7.00 ^b^	4	5.5	9.50 ^a^
Turnip extract at 0 ppm	4.75 ^a^	1.75 ^c^	6.50 ^b^	4.5	4.75	9.25 ^a^
Turnip extract at 150 ppm	4.00 ^b^	1.00 ^c^	5.00 ^c^	3.25	5	8.25 ^b^
Turnip extract at 300 ppm	5.00 ^a^	1.50 ^c^	6.50 ^b^	4.25	5	9.25 ^a^
Turnip extract at 450 ppm	4.75 ^a^	3.25 ^a^	8.00 ^a^	4.25	5.75	10.00 ^a^
*p*-value	0.03	0.001	0.001	0.67	0.8	0.01
SEM	0.112	0.205	0.255	0.266	0.267	0.176

* Means within each column without common superscripts are significantly different at *p* < 0.05.

**Table 6:** Mean ±SEM of organ weights related to immunity of Ross broilers at day 42, for birds supplemented with different levels of *Brassica rapa* extract at 0–450 ppm or Virginiamycin from weeks 1–6 *.

	Spleen g	Cloacal Bursa g
Virginiamycin at 0.2 g/kg	4.50 ^a^	4.00
Turnip extract at 0 ppm	3.00 ^b^	3.25
Turnip extract at 150 ppm	3.25 ^ab^	3.25
Turnip extract at 300 ppm	3.25 ^ab^	3.75
Turnip extract at 450 ppm	3.25 ^ab^	3.50
*p*-value	0.10	0.15
SEM	0.198	0.114

* Means within each column without common superscripts are significantly different at *p* < 0.05.

**Table 7:** Means ±SEM of cecum microbiota parameters at day 42, for Ross 308 broilers supplemented with different levels of *Brassica rapa* extract at 0–450 ppm or Virginiamycin from weeks 1–6 *.

	Coliform Bacteria cfu/g	Gram-Negative Lactose Bacteria cfu/g
Virginiamycin at 0.2 g/kg	2.42 ^a^	1.03 ^c^
Turnip extract at 0 ppm	1.99 ^bc^	2.27 ^a^
Turnip extract at 150 ppm	2.07 ^ab^	1.96 ^ab^
Turnip extract at 300 ppm	1.64 ^cd^	1.62 ^abc^
Turnip extract at 450 ppm	1.27 ^d^	1.48 ^bc^
*p*-value	0.001	0.02
SEM	0.1	0.13

* Means within each column without common superscripts are significantly different at *p* < 0.05.

## Data Availability

Data is available at Figshare, https://figshare.com/articles/dataset/alirezalatestdata_pdf/14211032.

## Appendix A

**Table A1:** Weekly performance mean at ±SEM of Ross 308 broilers fed the different levels of *Brassica rapa* extract from weeks 1–6 of age *.

	Weight Gaing/Bird	Feed Intakeg/Bird	Feed Conversion Ratio	Economic Index	Weight Gaing/Bird	Feed Intakeg/Bird	Feed Conversion Ratio	Economic Index
	1st week of age, d 1–7				4th week of age, d 22–28			
Virginiamycin at 0.2 g/kg	165.4 ^a^	174.3	1.06 ^b^	224.75 ^a^	537.5 ^cd^	769.7	1.43 ^a^	537.3 ^bc^
Turnip extract at 0 ppm	169.5 ^a^	73.3	1.03 ^b^	236.82 ^a^	605.1 ^ab^	787.2	1.30 ^ab^	676.0 ^a^
Turnip extract at 150 ppm	171.7 ^a^	177.8	1.03 ^b^	237.34 ^a^	513.0 ^d^	742.8	1.45 ^a^	507.1 ^c^
Turnip extract at 300 ppm	158.3 ^a^	170.3	1.07 ^b^	210.56 ^a^	569.4 ^bc^	700.9	1.22 ^b^	662.5 ^ab^
Turnip extract at 450 ppm	142.4 ^b^	172.8	1.21 ^a^	168.25 ^b^	638.6 ^a^	740.3	1.16 ^b^	791.8 ^a^
*p*-value	0.008	0.43	0.001	0.005	0.001	0.37	0.013	0.002
Standard Error of Mean	3.18	1.21	0.0195	7.504	12.49	13.9	0.0346	29.12
	2nd week of age, d 8–14				5th week of age, d 29–35			
Virginiamycin at 0.2 g/kg	264	345.1	1.31 ^a^	291.3	593.7	1066 ^a^	1.80 ^a^	474.3^b^
Turnip extract at 0 ppm	261.5	318.8	1.22 ^ab^	310.1	653.7	1102 ^a^	1.68 ^ab^	555.2 ^ab^
Turnip extract at 150 ppm	280.1	312.4	1.11 ^b^	360.8	650.6	1033 ^ab^	1.59 ^bc^	588.9 ^ab^
Turnip extract at 300 ppm	284.2	319.4	1.13 ^ab^	363.6	689.7	1031 ^ab^	1.50 ^b^	663.0 ^a^
Turnip extract at 450 ppm	296.8	308.5	1.04 ^b^	408.8	663.4	987 ^b^	1.48 ^b^	637.0 ^a^
*p*-value	0.22	0.16	0.04	0.08	0.07	0.04	0.008	0.03
Standard Error of Mean	5.45	5.049	0.031	15.06	11.19	12.88	0.036	21.54
	3rd week of age, d 15–21				6th week of age, d 36–42			
Virginiamycin at 0.2 g/kg	413.7 ^a^	587.8	1.42 ^b^	416.1 ^a^	408.7 ^b^	714.8	1.76	336.5
Turnip extract at 0 ppm	402.7 ^a^	568.1	1.41 ^b^	408.4 ^a^	382.5 ^b^	806.1	2.14	263.5
Turnip extract at 150 ppm	425.8 ^a^	584.5	1.37 ^b^	443.3 ^a^	637.2 ^a^	877.3	1.42	698.5
Turnip extract at 300 ppm	347.8 ^b^	562.2	1.63 ^a^	311.4 ^b^	574.8 ^ab^	788.9	1.44	626.9
Turnip extract at 450 ppm	338.2 ^b^	545.8	1.61 ^a^	300.3 ^b^	374.2 ^b^	662.4	1.96	332.6
*p*-value	0.001	0.07	0.009	0.001	0.05	0.08	0.09	0.1
Standard Error of Mean	9.421	5.408	0.03334	15.92	37.46	26.91	0.102	64.95

* Means within each column without common superscripts are significantly different at *p* < 0.05.
